# Risk of Drug-induced Movement Disorders with Newer Antipsychotic Agents

**DOI:** 10.5334/tohm.695

**Published:** 2022-06-08

**Authors:** George T. Kannarkat, Stanley N. Caroff, James F. Morley

**Affiliations:** 1 Philadelphia Parkinson’s Disease Research, Education and Clinical Center, Corporal Michael J. Crescenz Department of Veterans Affairs Medical Center, Philadelphia, PA, USA; 2 Department of Neurology, University of Pennsylvania, Philadelphia, PA, USA; 3 Department of Psychiatry, University of Pennsylvania, Philadelphia, PA, USA

**Keywords:** drug-induced movement disorders, tardive syndromes, antipsychotics, drug-induced parkinsonism, neuroleptic

## Abstract

**Background:**

The last decade has seen development of numerous novel antipsychotic drugs with unique mechanisms including long-acting formulations for clinical use. A comparative assessment of these new drugs with each other and previous antipsychotics have not been performed with regards to risk for drug-induced movement disorders (DIMD).

**Methods:**

Medline was searched from January 2010 to February 2022 for primary research articles and review articles in English using the search terms “extrapyramidal” and “tardive” with individual drug names of novel antipsychotics.

**Results:**

We identified articles describing the risk of DIMD with 6 novel antipsychotics, 4 novel formulations, and 3 experimental antipsychotics. Both short- and long-term data generally showed comparable to lower risk of DIMD with novel antipsychotics and recent long-acting formulations compared to previously marketed antipsychotics.

**Discussion:**

Several novel antipsychotics, particularly lumateperone and pimavanserin, show promise in being able to treat psychosis while reducing the risk of DIMD. Long-acting paliperidone may reduce risk of DIMD while other long-acting injectable formulations of SGA have similar risk of DIMD compared to oral formulations. New drug targets for treating psychosis without dopamine blockade also show promise.

## Introduction to Drug-Induced Movement Disorders (DIMD)

The association of antipsychotics with DIMD has been attributed primarily to their dopamine 2 receptor (D2R) blocking properties [1]. Acute DIMD refers to early-onset and reversible phenomena including akathisia, dystonia, parkinsonism, and tremor [2]. Tardive DIMD have a variable presentation, most commonly including stereotypy or chorea, but also dystonia, akathisia, myoclonus, tics, and tremor occurring at least weeks to months after drug exposure and often persisting despite drug cessation [1,3]. The prevalence of tardive syndromes among antipsychotic-treated patients is estimated to be between 20–30%, with annual incidence significantly increased by exposure to first-generation antipsychotics (FGAs) versus second-generation antipsychotics (SGAs) of 5.4–7.7% versus 0.8–3% (p < 0.0001), respectively, as well as by patient age [4,5]. Although SGA use has reduced the incidence of DIMD compared to FGAs, the expansive marketing and prescription of SGAs may increase the absolute number of individuals at risk [6]. Understanding how novel antipsychotics influence risk of the development of acute and tardive DIMD is critical to reducing patient harm. Although comparisons between reported studies are constrained by differences in design, methodology, and sample populations, we herein descriptively review data on acute and tardive DIMD associated with novel antipsychotics that have received Food and Drug Administration (FDA) approval since 2010 in addition to drugs in development.

## Methods

In this review, we restricted medications discussed to antipsychotic agents approved by the FDA since 2010: brexpiprazole, cariprazine, lumateperone, lurasidone, pimavanserin, olanzapine-samidorphan (O-S), long-acting injectable (LAI) risperidone, LAI aripiprazole, LAI paliperidone, transdermal asenapine, and inhaled loxapine. The terms in the Medline database searched from January 2010 to February 2022 and the identified additional articles through reference review are indicated in Supplementary Table 1. Articles reporting original interventional or observational studies addressing the incidence, prevalence or risk of antipsychotic-related DIMD were included in the review. Articles unavailable in English or exclusively focusing on preclinical data were excluded. Case reports, non-systematic reviews and position/editorial papers were not cited directly unless reporting relevant data. Sample sizes and results of statistical testing demonstrating significant differences between groups in cited studies are indicated within the text when reported in original studies.

**Supplementary Table 1:** Search terms used and number of results and articles reviewed.


SEARCH TERM	NUMBER OF RESULTS	EXCLUDED RESULTS	NUMBER OF ADDITIONAL ARTICLES IDENTIFIED THROUGH REFERENCE REVIEW	NUMBER OF ARTICLES SELECTED FOR REVIEW

“brexpiprazole” AND “tardive”	7	2	3	10

“brexpiprazole” AND “extrapyramidal”	28	7	25	46

“cariprazine” AND “tardive”	5	2	2	5

“cariprazine” AND “extrapyramidal”	54	3	10	61

“lumateperone” AND “tardive”	2	0	5	7

“lumateperone” AND “extrapyramidal”	8	2	0	6

“lurasidone” AND “tardive”	3	0	0	3

“lurasidone” AND “extrapyramidal”	53	13	5	45

“pimavanserin” AND “tardive”	1	0	0	1

“pimavanserin” AND “extrapyramidal”	6	3	2	5

“olanzapine” “samidorphan” AND “tardive”	0	0	0	0

“samidorphan” AND “tardive”	0	0	0	0

“olanzapine” “samidorphan” AND “extrapyramidal”	0	0	0	0

“samidorphan” AND “extrapyramidal”	0	0	0	0

“olanzapine” “samidorphan”	34	8	2	28

“long-acting” “risperidone” AND “tardive”	15	5	10	20

“long-acting” “risperidone” AND “extrapyramidal”	106	31	0	75

“long-acting” “aripiprazole” AND “tardive”	4	0	0	4

“long-acting” “aripiprazole” AND “extrapyramidal”	21	6	2	17

“long-acting” “paliperidone” AND “tardive”	10	2	0	8

“long-acting” “paliperidone” AND “extrapyramidal”	52	10	0	42

“transdermal” “asenapine” AND “tardive”	0	0	0	0

“transdermal” “asenapine” AND “extrapyramidal”	1	0	6	7

“inhaled” “loxapine” AND “extrapyramidal”	4	0	5	9

“inhaled” “loxapine” AND “tardive”	0	0	0	0


## Incidence of DIMD with Novel Antipsychotics

### a. Brexpiprazole

#### i. Mechanism and Unique Features

Brexpiprazole’s mechanism of action in treatment of psychosis and treatment-resistant depression (TRD) is primarily through partial agonism at D2Rs and 5-HT_2A_Rs with antagonism at 5-HT_2A_Rs [7]. Brexpiprazole has less partial agonism at the D2R relative to aripiprazole but has higher 5-HT2A antagonism which may explain its lower likelihood of inducing DIMD. Aripiprazole’s higher potency in inhibiting acetylcholinesterase activity may also play a role in its propensity to cause more DIMD relative to brexpiprazole [8]. It was approved for use in schizophrenia and as adjunctive treatment of depression in 2015 [9].

#### ii. Incidence of DIMD

The most common acute DIMD associated with brexpiprazole is akathisia occurring within three weeks of drug initiation [9]. In two double-blind, randomized control trials (DBRCTs) evaluating brexpiprazole for treatment of schizophrenia (n = 636, 674), there was no significant difference in acute DIMD as measured by multiple scales, including the Simpson-Angus Scale (SAS), Barnes Akathisia Rating Scale (BARS), Abnormal Involuntary Movement Scale (AIMS), and the Drug-Induced EPS Scale (DIEPSS), at doses up to 4mg/day after 6 weeks relative to baseline or placebo [9,10]. Other studies (n = 282, 379) have shown higher rates of akathisia relative to placebo (6.5–13.5% versus 1.0–7.1%) at doses of brexpiprazole greater than 2 mg/day [11,12]. A six-week phase III trial (n = 459) for acute schizophrenia episodes in Japan demonstrated 9–11% of individuals in treatment groups needed medication for parkinsonism relative to 4% in the placebo group while akathisia (1.7–5.3% versus 6.9%) was less frequent in the treatment groups [13]. A 52-week open-label Japanese trial (n = 150) for maintenance treatment of schizophrenia demonstrated that the rates of akathisia and tremor to be 8.5% and 4.3%, respectively [11]. Another one-year DBRCT (n = 524) for maintenance treatment of schizophrenia demonstrated no significant increase in acute DIMD based on motor rating scales with equal rates of akathisia (1.0%) but higher rates of tremor (3.0% versus 1.0%) relative to placebo [14]. A meta-analysis of three brexpiprazole (2–4 mg/day) RCTs (n = 1444) demonstrated no significant differences in SAS, BARS, or reported DIMD compared to placebo [15].

Similarly, in two phase III DBRCTs for TRD (n = 353, 677), brexpiprazole showed minimal but significant increases in the BARS (0.18 vs 0.01 points, p = 0.0001), mixed changes in AIMS (0.08 vs 0.0 points, p = 0.014; 0.03 vs 0.04, p = 0.87) and SAS (0.18 vs –0.02, p = 0.004; 0.12 vs 0.0, p = 0.053), and no difference in DIEPSS scores relative to placebo with one individual withdrawing due to drug-induced akathisia [12,16]. Incidence of akathisia ranged from 3–13% in these studies in a dose-dependent manner relative to placebo 1.0–2.3% [12,16]. Other studies (n = 49–1044) have shown comparable rates of acute DIMD in both short-term and long-term studies [17,18].

In a 52-week open-label extension of phase III trials for schizophrenia, only one of 1072 individuals developed TD, classified as moderately severe [18]. One case report demonstrated copulatory dyskinesia in association with brexpiprazole use [19]. While most data suggests that DIMD from brexpiprazole is not severe, one case report described severe parkinsonism [20]. One case report using brexpiprazole in the treatment of Parkinson’s disease psychosis demonstrated no AE [21]. Overall, akathisia is the most common DIMD with brexpiprazole but rates of DIMD in general are low.

#### iii. Comparison to previous SGAs

Relative to adjunctive aripiprazole use (n = 353, 677), brexpiprazole showed lower SAS and BARS scores and a lower incidence of akathisia in TRD [12,16]. In an open-label RCT for schizophrenia comparing aripiprazole (n = 33) to brexpiprazole (n = 64), DIMDs were twice as likely in the aripiprazole-treated group, most commonly akathisia (21.2% versus 9.4%), without significant differences in SAS or BARS [22]. In a small cohort of 37 Japanese individuals with schizophrenia or schizoaffective disorders, switching from other SGAs to brexpiprazole significantly improved scores by 1 point on average on the DIEPSS (p = 0.008) after eight weeks. Conversion of patients to brexpiprazole did not demonstrate any significant increase in DIMD [23]. A systematic review (n = 3925) comparing aripiprazole to brexpiprazole demonstrated no significant difference in the rates of akathisia or other DIMD [24]. In terms of akathisia, quetiapine, brexpiprazole, and ziprasidone have the highest number needed to harm (NNH) at 188, 112, and 72 in a study comparing SGAs [25]. Compared to other SGAs, brexpiprazole has a more favorable rate of DIMD in general but is prone to causing akathisia [26].

### b. Cariprazine

#### i. Mechanism and Unique Features

Relative to aripiprazole and brexpiprazole, cariprazine has a tenfold higher affinity for D3Rs with partial agonism at D2Rs and 5-HT_1A_Rs [27]. The D3R is preferentially expressed in brain regions, such as the ventral striatum, that regulate reward and motivation rather than motor control which could explain its proposed efficacy in the treatment of negative symptoms in schizophrenia [28]. It is FDA approved for the treatment of schizophrenia and bipolar disorder (BPD) [29].

#### ii. Incidence of DIMD

Safety and proof of concept trials (n = 392) with cariprazine demonstrated adverse events of total acute DIMD, tremor, and akathisia of 6–10%, 3–6%, and 9–10%, respectively [30]. A phase II DBCRT study (n = 732) in acute schizophrenia episodes for eight weeks demonstrated acute parkinsonism and akathisia in 8–10% and 11–15%, respectively, (1.5–2 times of placebo) with a 2% DIMD-related discontinuation rate [31]. Using higher doses (up to 9 mg/day) of cariprazine in a phase III trial (n = 446) demonstrated rates of acute akathisia, total DIMD, parkinsonism, use of anti-DIMD medications, and tremor of up to 17%, 10%, 17%, 24% and 5%, respectively, with no attributable discontinuation [32]. While the rates of DIMD were higher than placebo, they were only increased in the 6–9 mg/day group relative to the 3–6 mg/day group for total acute DIMD (10% versus 5%), acute parkinsonism (17% versus 9%), and use of anti-DIMD medications (24% versus 16%) [32]. Other phase III DBRCTs (n = 461) and open-label studies (n = 60) including studies up to 72 weeks have shown similar rates of acute DIMD [33,34]. Discontinuation rates in these trials were less than 2% for acute akathisia, total DIMD, or parkinsonism [33,34,35,37]. In a long-term 48-week trial (n = 679), rates of acute akathisia, parkinsonism, and total DIMD ranging from 13–23%, 11–15%, and 6.5–8.1% were observed when stratified by modal daily dose ranges [36]. Interestingly, akathisia and parkinsonism showed an inverse relationship to dose which could be due to limitations in uptitration based on tolerability [36]. Similarly, pooled analysis of cariprazine DBRCTs (n = 2048, 2193) for acute schizophrenia episodes using doses from 1.5–12 mg/day showed dose-dependent rates of akathisia, total DIMD, and parkinsonism of 7.8–18%, 6.0–11.3%, and 10.3–17.4%, respectively [38,39]. Interestingly, use of cariprazine for schizophrenia in an elderly subgroup (n = 17 elderly, n = 83 total) was not associated with akathisia as an adverse event in one small study [40].

In a DBRCT (n = 819) evaluating cariprazine as an adjunctive therapy for TRD, akathisia occurred in 6.6% and 22.3% in low and high dose treatment groups compared to 2.3% in placebo [41]. Parkinsonism occurred in less than 3% of treated individuals [41]. Acute DIMD occurred in 3.0%, 6.2% and 11.0% of placebo, low and high-dose treated groups, respectively, with discontinuation in 0.4%, 0.7%, and 1.1% [41]. Use of cariprazine in trials (n = 238, 403, 233) for acute manic or mixed episodes in BPD showed similar rates of acute DIMD as studies in schizophrenia and depression [42,43,44]. Pooled analysis of studies using cariprazine in bipolar depression and another pooled analysis of three RCTs (n = 1065, 1407) in bipolar mania showed a dose-dependent increase in akathisia (2.2–9.6% versus 0–2.4%; NNH = 19) and other DIMD (3.0–29.2% versus 2.1–11.5%; NNH = 43) relative to placebo with discontinuation due to these AEs in 3% or less of individuals [45,46].

In pooled analyses (n = 1407), TD was rare in the short-term, occurring in less than 0.5% of individuals over 7–9 weeks [46]. A systematic review of nine RCTs (n = 4324) in patients with BPD, schizophrenia, and MDD, cariprazine had a significantly higher risk of akathisia (relative risk [RR] = 3.92, 95 % CI 2.83–5.43) and tremor (RR 2.41, 95% CI 1.53–3.79) compared to placebo [47].

#### iii. Comparison to previous SGAs

In a phase II trial (n = 732) for acute exacerbation of schizophrenia, cariprazine had lower acute DIMD than the risperidone (4 mg/day) group [31]. Another phase III trial (n = 461) for negative symptoms in schizophrenia and a pooled RCT analysis (n = 2193) showed that rates of akathisia were slightly higher in the cariprazine group (8%) compared to the risperidone group (5%) whereas rates of total DIMD and parkinsonism were similar to slightly higher in the risperidone group (4–7% versus 6–10%) with similar rates of discontinuation (1–2%) [33,38]. Use of 6 mg/day (n = 157) but not 3 mg/day (n = 155) cariprazine was associated with increased akathisia relative to 10 mg/day (n = 152) aripiprazole [32]. In a pooled analysis (n = 2193) of schizophrenia trials, rates of DIMD were higher for risperidone 4 mg/day (27.1%) compared to cariprazine 1.5–12mg/day (17.4%) but not aripiprazole 10 mg/day (5.9%) while akathisia rates were higher for cariprazine (11.3%) relative to risperidone (8.6%) or aripiprazole (7.2%) [38]. Rates of parkinsonism were similar in the risperidone group while half in the aripiprazole group relative to cariprazine [38]. NNH for akathisia is estimated to be ~30 for cariprazine, 34 for quetiapine, and 15 for lurasidone (n = 1407) [46]. A network meta-analysis (n = 4324) of multiple RCTs showed that there was no significant difference in overall rates of DIMD comparing aripiprazole, quetiapine, cariprazine, and lurasidone but surface under cumulative ranking curves (SUCRA) demonstrate reduced risk of DIMD for aripiprazole (overall DIMD 0.57, akathisia 0.51) and cariprazine (overall DIMD 0.45, akathisia 0.38) relative to quetiapine (overall DIMD 0.28) and lurasidone (overall DIMD 0.22, akathisia 0.13) [48]. Cariprazine may be more prone to causing akathisia, particularly early in treatment, relative to aripiprazole and risperidone while overall DIMD rates are intermediate among SGAs.

### c. Lumateperone

#### i. Mechanism and Unique Features

Lumateperone is a first-in-class medication that simultaneously acts at serotonergic, dopaminergic, and glutamatergic receptors [49]. It was FDA approved for use in schizophrenia in 2020 but is also under investigation for mood disorders [49,50]. At 5-HT_2A_Rs, it acts as an antagonist with 60-fold higher affinity than at D2Rs, where it acts pre-synaptically as a partial agonist and post-synaptically as an antagonist, which is proposed to reduce its risk of causing DIMD [49]. By contrast, other SGAs like risperidone and olanzapine have a 12:1 affinity for binding of D2Rs and 5-HT_2A_Rs. By inhibiting a serotonin transporter, it also increases phosphorylation of GluN2B receptors in mesolimbic pathways. The unique mechanism of this drug suggests that it could be useful at low doses for sleep and agitation reduction with antipsychotic and antidepressant effects at higher doses [50].

#### ii. Incidence of DIMD

A phase II DBRCT (n = 335) using doses of 60–120 mg/day of lumateperone in acute exacerbation of schizophrenia demonstrated AEs of akathisia in 2% of subjects, which was comparable to placebo. There were no significant change in SAS, AIMS, or BARS over 28 days [51]. Indeed, phase III DBRCTs (n = 450) and open label studies (n = 1481) with 40–60 mg/day confirm that the rates of acute DIMD are similar to placebo for as long as 12 months [52,53]. In pooled studies (n = 381, 1073), lumateperone showed similar rates of acute DIMD and use of medications to treat DIMD compared to placebo and lower rates than risperidone (4 mg/day) [54,55]. No clear reports of tardive syndromes associated with lumateperone have been published.

### d. Lurasidone

#### i. Mechanism and Unique Features

Lurasidone is approved for acute and maintenance treatment of schizophrenia and for treatment of bipolar depression [56]. It acts via potent antagonism at 5-HT_7_R and partial agonism at 5-HT1_A_Rs mediating cognitive and antidepressant effects while having significant affinity for 5-HT_2A_Rs and D2Rs [56].

#### ii. Incidence of DIMD

Short-term, six week, trials (n = 149, 285, 500) of lurasidone in a DBRCT for acute schizophrenia episodes showed dose-dependent total acute DIMD, parkinsonism, dystonia, and akathisia AE rates of 4–26%, 4–11%, 3–4%, and 2–24% [57,58,59,60]. Initiation of anticholinergic medications for DIMD was higher than placebo (13.7–29.0% versus 7.9–18.0%) [57,58]. Discontinuation rates due to these AEs were less than 3%, and were mainly attributable to akathisia [57,58]. However, there were generally not significant changes in SAS, BARS, and AIMS except at 120 mg/day doses [57,60]. One RCT (n = 285) in acute schizophrenia episodes showed higher AIMS (1.7–4.9% versus 2.4%), SAS (5.9–6.6% versus 2.4%), and BAS (2.5–4.0% versus 0%) scores relative to placebo [58].

Six-week RCTs (n = 339, 348, 356) in bipolar depression using lurasidone (20–120 mg/day) demonstrated dose-dependent AE rates for akathisia, tremor, and total DIMD of 7.7–14.1%, 8.2%, and 4.9–15.3% which were higher than placebo [61,62,63]. Change in BAS, SAS, and AIMS were generally minimal and not significant but one study showed a significant increase in the BAS by 0.1 [61,62,63]. Anticholinergic medications were used by 6.8–11% in the lurasidone group versus 3.5–4% for placebo group in these studies [62,63]. A DBRCT (n = 211) in major depressive disorder with hypomanic features showed lower rates of parkinsonism (2.8%) and akathisia (3.7%) than similar studies in bipolar depression [64]. A year-long open label study (n = 199) of lurasidone in maintenance BPD treatment demonstrated akathisia in 30.7% and parkinsonism in 7.5% of treated individuals with 4% of individuals discontinuing treatment for akathisia [65].

Longer term trials, including DBRCTs (n = 148, 629) and open-label extensions over 4–12 months in schizophrenia, have shown relatively similar rates of DIMD including akathisia (8.1–14%), parkinsonism (1.4–4.3%), dystonia (1.4–3.1%), and tremor (2.0–3.1%) [66,67]. Discontinuation due to akathisia was 1% or less [66,67]. BARS, SAS, and AIMS scores did not demonstrate significant changes [67]. A meta-analysis (n = 1608) of three DBRCTs for lurasidone in schizophrenia demonstrated higher rates of akathisia (risk ratio = 2.999; 95% CI 1.64–5.45) and dystonia (risk ratio = 6.013; 95% CI 1.51–23.99) compared to placebo [68].

A 24-week open-label extension trial (n = 813) for lurasidone’s adjunctive use in bipolar depression showed rates of overall DIMD and akathisia as high as 10.7% and 8.1%, respectively, with these AEs found more commonly in adjunctive therapy rather than monotherapy arms [69]. Another 28-week open label extension trial (n = 413) showed akathisia and parkinsonism in 18.2% and 7.3%, respectively [70]. Discontinuation rate of lurasidone due to akathisia was less than 3% in these studies [69,70]. A DBRCT (n = 496) with placebo control, using a 20-week stabilization phase and subsequent 28 week treatment period in BPD showed similar rates of DIMD as other long-term studies [71]. Case reports have identified individuals with TD, drug-induced parkinsonism, rabbit syndrome, and laryngospasm with lurasidone-exposure [72,73,74,75,76].

#### iii. Comparison to previous SGAs

In a 12-month trial (n = 427) in maintenance treatment for schizophrenia, similar rates of parkinsonism (4–5%) and tremor (3%) but significantly higher rates of akathisia (14.3% versus 7.9%; NNH = 16, p < 0.05) were observed for lurasidone (40–120 mg/day) and non-significantly higher rates of dystonia for risperidone (2–6 mg/day, 3.1% versus 5.9%) [66]. Six-week trials (n = 478, 488) for acute schizophrenia episodes using extended-release quetiapine 600 mg or olanzapine 15 mg daily in a separate arm relative to lurasidone demonstrated increased rates of reported DIMD, parkinsonism, and akathisia in the lurasidone arm [77,78]. A 12-month non-inferiority study (n = 292) comparing quetiapine XR (200–800 mg/day) to lurasidone (40–160 mg/day) for maintenance therapy in schizophrenia similarly demonstrated lower rates of DIMD (11.9–21.4% versus 3.5%) and akathisia (10.7–12.6% versus 2.4%) in the quetiapine arm [79]. A systematic review (n = 1223) of three DBRCTs comparing lurasidone, olanzapine, and quetiapine-XR in bipolar depression demonstrated significantly increased risk for quetiapine-XR causing acute DIMD (RR = 3.11, 95% CI 1.36–7.09) while both quetiapine-XR (RR = 3.71, 95% CI 1.26–10.95) and lurasidone (RR = 2.04, 95% CI 1.03–4.04) significantly increased risk for akathisia relative to placebo [80]. A short 21-day DBRCT (n = 301) comparing lurasidone (120 mg/day) to ziprasidone (160 mg/day) demonstrated comparable low rates of DIMD including akathisia [81]. In a comparative efficacy study (n = 43,049), lurasidone (OR 2.46, 95% CI 1.55–3.72) had a risk of DIMD similar to antipsychotics such as risperidone (OR = 2.09, 95% CI 1.54–2.78) and paliperidone (OR = 1.81, 95% CI 1.17–2.69) but higher than clozapine (OR = 0.3, 95% CI 0.12–0.62) relative to placebo [82]. A meta-analysis of fourteen studies (n = 6221) in bipolar depression demonstrated no difference in rates of DIMD among lurasidone, quetiapine, aripiprazole, olanzapine, and ziprasidone [83]. Lurasidone seems to have comparable risk of causing akathisia and other DIMD relative to previously marketed SGAs.

### e. Pimavanserin

#### i. Mechanism and Unique Features

Pimavanserin is a 5-HT_2A_R inverse agonist and was approved for use in Parkinson’s disease (PD) psychosis (PDP) in 2016 at a dose of 34 mg daily [84]. Because of its unique selectivity for this receptor, located on multiple types of excitatory neurons, it has benefit in psychosis without causing anti-dopaminergic side effects [85]. Decreasing signaling through the 5-HT_2A_ receptor may impact psychosis through decreasing dopamine release in the nucleus accumbens and in ventral tegmental neurons that project to frontal cortical and limbic regions [86].

#### ii. Incidence of DIMD

In placebo-controlled DBRCTs (n = 60, 199) in PDP, AEs were similar between placebo and treatment groups [86,87]. Use of pimavanserin in conjunction with subtherapeutic doses of risperidone or haloperidol in psychosis in chronic schizophrenia did not show differences in motor AEs or as measured by SAS or BARS compared to placebo (n = 423) [84]. Very preliminary evidence (n = 403) for efficacy and similar tolerability in adjunctive treatment of negative symptoms in schizophrenia also exists without increase in DIMD while other studies are ongoing [88,89,90,91]. No studies have specifically cited or examined the risk of tardive syndromes with pimavanserin.

#### iii. Comparison to previous SGAs

No studies were identified that have evaluated the risk of DIMD in pimavanserin relative to SGAs.

### f. Olanzapine-samidorphan (O-S)

#### i. Mechanism of action

Olanzapine is an SGA that antagonizes 5-HT_2A/2C_Rs, 5-HT_6_Rs, D1-4Rs, histamine-1 receptors (H1Rs) and α_1_-adrenergic receptors and has been associated with weight gain and propensity for development of metabolic syndrome [92]. Samidorphan has been shown to alter glucose homeostasis and has been used clinically to mitigate weight gain caused by olanzapine [92]. O-S was approved in 2021 for treatment of schizophrenia and BPD [92].

#### ii. Incidence of DIMD

In a four-week DBRCT (n = 401) for acute exacerbations of schizophrenia, there was no significant difference between AIMS, BARS, or SAS scores compared to olanzapine alone [93]. In the two-week follow-up, rates of parkinsonism (3.7% vs 4.5%), akathisia (6.0% versus 4.5%), and dyskinesia (1.5% vs 0.8%) were comparable between the O-S versus the olanzapine group and lower than placebo [93]. The safety profile with regard to DIMD was similar in other studies (n = 42, 234, 277 309, 561) with evaluation up to 52 weeks with no discontinuation of drug related to DIMD AEs [94,95,96,97,98].

## 4. Incidence of DIMD with Alternative Formulations of Antipsychotics

### a. LAI antipsychotics and other novel formulations

LAI antipsychotics were developed to improve poor medication adherence observed with oral formulations [99]. Studies looking at pooled data comparing oral versus LAI formulations have had mixed results with some showing no difference in DIMD whereas others have suggested higher rates with LAIs [100,101,102]. We review below evidence regarding incidence of acute DIMD and tardive syndromes in recently developed LAIs and other novel drug formulations.

#### i. LAI Risperidone

##### 1. Mechanism and Unique Features

LAI risperidone was the first SGA available in depot form and is now available in intramuscular and subcutaneous formulations of varying dosages and half-lives [103]. LAI risperidone has lower peak levels and fluctuations in levels than oral formulations [104]. Risperidone is an antagonist at D2Rs greater than D1Rs and has inverse agonist activity at 5-HT_2A/2C_Rs [105].

##### 2. Incidence of DIMD

Both short (n = 49, 264, 804) and long term (up to one year) studies (n = 215) in acute and maintenance treatment of schizophrenia and BPD have demonstrated that rates of acute DIMD, ranging from 3–23% were not significantly different between LAI risperidone and placebo and there were low not significantly different rates of discontinuation (<1%) due to DIMD [103,106,107,108]. Interestingly, a 3 year study (n = 108) transitioning individuals from oral antipsychotics to LAI risperidone demonstrated reductions in TD and acute DIMD only in individuals with what was diagnosed as concurrent dopamine supersensitivity psychosis, suggesting that switching to LAIs may reduce the incidence of acute DIMD and TD in a subset of individuals [109]. An open-label 52-week study (n = 652) of LAI risperidone demonstrated ~1% rate of persistent TD which is comparable to oral SGAs [110]. A case of atypical TD involving the cricothyroid musculature in LAI risperidone has been reported [111]. Meta-analyses and systematic reviews comparing LAI versus oral risperidone showed no difference in DIMD [112,113].

#### ii. LAI Paliperidone palmitate

##### 1. Mechanism and Unique Features

Paliperidone, a metabolite of risperidone, has 1-, 3-, and 6-month LAI formulations [114]. Paliperidone is pharmacologically similar to risperidone with a few exceptions including less protein binding in plasma (~74%), ~60% direct renal excretion, and higher affinity for 5-HT_2A_Rs [105].

##### 2. Incidence of DIMD

A 12-week phase II DBRCT (n = 197) showed increased parkinsonism relative to placebo (8% versus 1%) but no other increase in DIMD [115]. A larger phase III DBRCT (n = 514) with the monthly formulation showed similar rates of DIMD, most commonly parkinsonism (5–6%), to placebo and decreased use of anti-DIMD medications from 30% before to 6% after the double-blind period but no significant change in SAS, BARS, or AIMS scales [114]. Another 12-week DBRCT (n = 305) with the 3-month formulation showed increased rates of akathisia, 4%, relative to placebo, 1%, but no difference in other rates of DIMD [116]. Other studies (n = 197–951) in schizophrenia and schizoaffective disorder with LAI paliperidone also showed low rates of DIMD [117,118]. These results are similar to studies (n = 305, 514) with oral paliperidone and one pooled analysis (n = 3121) suggest relatively decreased DIMD rates with LAI paliperidone (anti-DIMD medication 17% versus 12%, p = 00035; BARS –0.09 versus –0.03, p = 0.023; SAS –0.04 versus 0.0, p < 0.0001) [114,116,119].

A pooled analysis (n = 3743) looking specifically at rates of TD showed low rates <0.2% regardless of administered formulation [120]. A case of unmasked parkinsonism has been reported with LAI paliperidone use persisting after drug cessation [121]. Case reports of tardive dystonia, TD, and parkinsonism, with LAI paliperidone are also found in the literature [122,123,124].

A 13-week open label trial (n = 452) in schizophrenia comparing LAI risperidone to LAI paliperidone showed higher rates of akathisia (19.7% versus 13%), tremor (17.9% versus 10.5%), and anti-DIMD therapy (46.2% versus 31.4%) in the risperidone group [125]. However, other RCTs and pooled analyses (n = 1214–2183) comparing LAI risperidone and LAI paliperidone showed <5% rates of DIMD without significant differences between groups [126,127]. LAI paliperidone had comparable minimal and non-significant changes in Extrapyramidal Symptom Rating Scale (ESRS) when compared to oral SGAs in smaller RCTs (n = 84, 444) [128,129]. One of these studies (n = 444) also suggested that LAI paliperidone had lower rates of DIMD compared to oral paliperidone [129]. Studies (n= 295) comparing LAI paliperidone to LAI aripiprazole showed low, comparable rates of DIMD [130].

#### ii. LAI Aripiprazole

##### 1. Mechanism and Unique Features

Aripiprazole monohydrate is a powder that is mixed with water for monthly intramuscular injections [131]. Aripiprazole lauroxil is administered from prefilled syringes ranging from 441 to 882 mg every 4–8 weeks intramuscularly [131]. Aripiprazole acts as a partial agonist at 5-HT_1A_Rs and as an antagonist at 5-HT_2A_Rs with a complex interaction at D2Rs [132]. At D2Rs, this drug acts as a partial antagonist at high extracellular dopamine concentrations and as a partial agonist at low extracellular dopamine concentrations [133].

##### 2. Incidence of DIMD

Short-term studies (n = 340, 623) using LAI aripiprazole in acute schizophrenia episodes did not show significant changes in motor scales but some studies showed a higher rate of akathisia relative to placebo (11% versus 4%) [134,135]. In a 52 week, placebo-controlled DBRCT (n = 710) of maintenance treatment of schizophrenia using LAI aripiprazole, rates of overall DIMD, akathisia, dyskinesia, dystonia, and parkinsonism were 14.9% vs 9.7%, 5.6% vs 6.0%, 0.7% vs 1.5%, 8.2% vs 3.0%, for treatment versus placebo, respectively, with no significant difference in change in AIMS, SAS, or BARS which is similar to data from other long term studies [136]. Frequency of anticholinergic therapy for DIMD in these studies was 16.7% versus 10.4% in placebo [135,136]. A meta-analysis (n = 986) comparing tolerability of LAI versus oral aripiprazole showed no difference in DIMD [112]. A systematic review (n = 4796) comparing various LAIs demonstrated that aripiprazole had a lower odds of DIMD compared to risperidone or paliperidone, but this effect was not significant due to high variability [130]. No reports of tardive syndromes related to LAI aripiprazole were found in the literature.

#### iv. Transdermal asenapine

##### 1. Mechanism and Unique Features

Asenapine is the first transdermal antipsychotic approved in 2019 by the FDA for treatment of schizophrenia. Patches are applied daily with equal hepatic and renal metabolism [137]. It antagonizes multiple receptors including 5-HT_2A/2B/2C/6/7_, D1/D2/D3Rs, and α_1_/α_2a/b/c_ adrenergic receptors with dose-dependent D2R occupancy [138].

##### 2. Incidence of DIMD

A phase III clinical trial (n = 253) showed no increase in motor scales assessing DIMD [139]. Data from this phase III DBRCT using sublingual asenapine showed akathisia and overall DIMD rates as high as 7.7% and 10%, respectively, during a 12–16-week open label period (n = 549) and 1.6% and 2.4%, respectively, during the 26-week double-blind phase (n = 253) relative to 0.8% in the placebo group [139]. Other studies (n = 80–532) in both schizophrenia and bipolar mania or mixed episodes showed similar rates of these AEs and minimal changes in motor rating scales [138]. No reports were found of tardive syndromes are associated with transdermal asenapine.

#### V. Inhaled loxapine

##### 1. Mechanism and Unique Features

Loxapine has existed as an oral formulation for over 40 years and also as an intramuscular formulation for the treatment of agitation [140]. The inhaled formulation (10 mg per inhalation) was approved by the FDA in 2012 for agitation in schizophrenia and mania and can achieve peak concentrations in 2–5 minutes with a half-life of 4 hours [140]. Structurally like clozapine it has postsynaptic antagonism at D2R and 5-HT_2A_R [140].

##### 2. Incidence of DIMD

In clinical trials (n = 129–344) for agitation in bipolar mania and schizophrenia, inhaled loxapine was not associated with any significant increase in DIMD [141]. Benztropine was given for one individual with acute akathisia and another with acute dystonia (jaw and oculogyric) which resolved symptoms in both cases. Oral formulations of loxapine have been associated with TD, tardive dystonia, and other DIMD at rates similar to SGAs [141]. However, the risk of tardive syndrome is likely rare with intended use of inhaled loxapine as rescue therapy.

## 5. Agents with novel mechanisms under investigation

Three novel classes of antipsychotics are currently under investigation whose mechanisms of action have the potential to obviate DIMD. Uloturant, a trace amine-associated receptor 1 (TAAR1) agonist, is currently in Phase 3 clinical trials for schizophrenia [142]. TAAR1s are G-protein coupled receptors expressed in multiple brain regions including the ventral tegmental area and the dorsal raphe nucleus, which allow for modulation of dopaminergic and serotonergic pathways improving positive schizophrenic symptoms and sleep [142]. While it has agonist activity at 5-HT_1A_Rs, it does not have antagonism at D2 or 5-HT_2A_Rs which is thought to improve its tolerability [142]. Early studies have not shown a risk of DIMD as expected from data in preclinical models with AEs most commonly being somnolence and GI symptoms [143].

Roluperidone is a high-affinity 5-HT_2A_ antagonist and σ2R antagonist [91]. Early trials have shown modest clinical efficacy without increased DIMD which is unsurprising given its lack of D2R antagonism [144]. Common side effects included headache, asthenia, and somnolence [91].

Finally, xanomeline is a selective M1 and M4 muscarinic receptor agonist [145]. While this drug has no significant direct binding to dopamine receptors, preclinical models demonstrate functional dopaminergic antagonism particularly in the ventral tegmental area [146]. Clinical trials have shown efficacy in schizophrenia with side effects mainly consisting of nausea, vomiting, gastrointestinal distress, and hypersalivation [145,147]. Notably, there were no DIMD-related AEs [145,147]. Ongoing studies will help better define the efficacy and tolerability of these medications.

## 6. Summary

The decision to select an antipsychotic for treatment depends on an informed risk-benefit analysis of each individual patient’s condition and needs, including consideration of efficacy and a range of potential side effects. Systematic reviews and meta-analyses show that in general, FGAs and SGAs, have comparable efficacy with differences that are on a continuum rather than discrete [148,149]. Research and development of antipsychotic drugs has largely been driven by the goal of eliminating adverse DIMD, especially irreversible tardive syndromes, while preserving antipsychotic effectiveness. The recent novel antipsychotics work through various mechanisms including greater serotonergic relative to dopaminergic antagonism, dose-dependent partial D2R agonism, and D3R antagonism [7,27,56]. These mechanisms allow for efficacy in psychosis while reducing incidence of DIMD (Table 2). Lumateperone and pimavanserin have a significantly lower risk of DIMD and tardive syndromes than previous antipsychotics, although relatively less data are currently available for these new agents. Relative to previous SGAs, brexpiprazole has a reduced risk of overall DIMD with increased risk of akathisia. Cariprazine and lurasidone have comparable rates of DIMD relative to previous SGAs with the former having a higher relative rate of akathisia.

**Table 2:** Summary of Novel Antipsychotics.


RISK OF OVERALL (ACUTE AND TARDIVE) DIMD	RISK OF TARDIVE SYNDROMES	RISK OF AKATHISIA	RISK OF PARKINSONISM	MECHANISM OF ACTION	FDA-APPROVED INDICATIONS	DRUG NAME

Less than previous SGAs [15,22,23]	Case reported [46]	Possibly increased relative to previous SGAs [66]	Less thar previous SGAs [15,23,153]	partial agonist at D2Rs and 5-HT_2A_Rs, antagonist at 5-HT_2A_Rs [7]	Schizophrenia and adjunct for depression [9]	Brexpiprazole

Similar to previous SGAs [31]	Less than 0.5% [31]	Slightly increased relative to previous SGAs [33,38,38,46,48]	Similar to previous SGAs [31]	partial agonist at D2R, D3R, and 5-HT_1A_R [27]	Schizophrenia and BPD [29]	Cariprazine

No increased risk relative to placebo; lower than previous SGAs [52,52,53,55]	Not Reported	No increased risk relative to placebo; lower than previous SGAs [52,53]	No increased risk relative to placebo; lower than previous SGAs [51,52,53]	Antagonist at 5-HT_2A_Rs, dopamine concentration dependent antagonism at D2Rs [56]	Schizophrenia [49,50,56]	Lumateperone

Two reports (acute akathisia, acute dystonia) [141]	Not reported	Single reported individual [141]	Not reported	postsynaptic antagonism at D2R and 5-HT_2A_R [140]	Agitation in schizophrenia and mania [140]	Inhaled Loxapine

Similar to previous SGAs [66,77,78,83]	Multiple case reports [72,73,74,75,76]	Similar to previous SGAs [77,80,82,83]	Similar to previous SGAs [66,77,80,81]	potent antagonist at 5-HT_2A_Rs, D2Rs, 5-HT_7_Rs, and partial agonist at 5-HT_1A_Rs [56]	Schizophrenia and bipolar depression [56]	Lurasidone

Equivalent to olanzapine [94,95,96,97,98]	Equivalent to olanzapine [94,95,96,97,98]	Equivalent to olanzapine [94,95,96,97,98]	Equivalent to olanzapine [94,95,96,97,98]	5-HT_2A/2C_R, 5-HT_6_R, D1-4R, H1R and a_1_-adrenergic receptor antagonist [92]	Schizophrenia and BPD [92]	Olanzapine-samidorphan

Not reported	Not reported	Not reported	Not reported	5-HT_2A_R inverse agonist [84]	Parkinson’s disease psychosis [84]	Pimavanserin

Similar to previous SGAs and oral formulations [112,113]	Similar to previous SGAs and oral formulations [110]	Similar to previous SGAs and oral formulations [112,113]	Similar to previous SGAs and oral formulations [112,113]	Antagonism at D2Rs greater than D1Rs and inverse agonist activity at 5-HT_2A/2C_Rs [105]	Schizophrenia and BPD [103]	LAI Risperidone

Similar to previous SGAs (possibly lower than oral formulations) [114,116,126]	Similar to previous SGAs [105,120,130]	Similar to previous SGAs [114,116,130]	Similar to previous SGAs [114,116,126,130]	Similar to risperidone with higher affinity for 5-HT_2A_R [105]	Schizophrenia and schizoaffective disorder; adjunct for BPD or major depressive disorder [114]	LAI Paliperidone

Similar to previous SGAs and oral formulations [112,130,134,135]	Similar to previous SGAs and oral formulations [112,130,134,135]	Similar to previous SGAs and oral formulations [112,130,134,135]	Similar to previous SGAs and oral formulations [112,130,134,135]	partial agonist at 5-HT_1A_Rs and as an antagonist at 5-HT_2A_Rs with a dose-dependent agonism/antagonism at D2Rs [132]	Schizophrenia, BPD, major depressive disorder [131]	LAI Aripiprazole

Similar to previous SGAs [138,139]	Not reported	Similar to previous SGAs [138,139]	Similar to previous SGAs [138,139]	antagonist at multiple receptors including 5-HT_2A/2B/2C/6/7_, D1/D2/D3Rs, and α_1_/α_2a/b/c_ adrenergic receptors with dose-dependent D2R antagonism [138]	Schizophrenia [137]	Transdermal Asenapine


DIMD = drug-induced movement disorder, FDA = Food and Drug Administration, LAI = long-acting injectable, SGA = second-generation antipsychotic.

LAI antipsychotics have also become increasingly used in the past 10 years to improve adherence [99]. LAI paliperidone may have lower rates of DIMD whereas LAI risperidone and LAI aripiprazole have similar rates compared to oral formulations. In general, alternate formulations of antipsychotics do not seem to change risk of DIMD. In the future, current drugs in the pipeline of research and development that further spare dopamine signaling suggest antipsychotic strategies that could promise separation from the intertwined and heretofore inevitable risk of acute and tardive DIMD.

Tardive syndromes with novel antipsychotics are reported to be overall rare in the trials reviewed here. However, there are major limitations that must be considered in the interpretation of this data. Most clinical trial designs include limited follow-up, at most a year; yet TD can take years to manifest, and thus may be underestimated in typical clinical trials. Furthermore, variable prior use of antipsychotics and inconsistent washout periods of prior antipsychotics complicates determination of the prospective risk of TD and other DIMDs in both placebo and intervention arms. Variations in age and sex of participants can impact incidence of DIMD and make comparisons between studies difficult. The studies reviewed had widely variable sample sizes (from 10s to 1000s) and variable reporting of statistical comparison of adverse events. Results from larger controlled trials likely should carry greater clinical impact. The outcome scales capturing DIMDs employed vary between studies, and even the best of these, the Simpson-Angus Scale, have been criticized as insensitive compared to instruments used by movement disorders neurologists. Finally other studies rely on the prescription of anticholinergic/antiparkinsonian medications as surrogate measures of DIMD which does not capture the underlying DIMDs.

Only further head-to-head comparison, multiyear real-world tolerability data, and assessment of DIMD with consistent neurologic examination compiled prospectively among initially drug-naïve individuals will give a more precise risk differential for these novel antipsychotics. While previous SGAs and novel antipsychotics have lower relative rates of DIMD compared to FGAs, increasing indications and widespread off-label use of SGAs and novel antipsychotics could lead to overall increased absolute prevalence of DIMD [150,151,152]. Understanding which of these medications have the greatest benefit-to-risk ratio is important to reduce the overall burden of DIMD.

## References

[1] Hauser RA, et al. Differentiating tardive dyskinesia: a video-based review of antipsychotic-induced movement disorders in clinical practice. CNS Spectr. 2020; 1–10. DOI: 10.1017/S109285292000200X PMC9249122 33213556

[2] Factor SA, et al. Recent developments in drug-induced movement disorders: a mixed picture. Lancet Neurol. 2019; 18: 880–890. DOI: 10.1016/S1474-4422(19)30152-8 31279747

[3] Caroff SN, Campbell EC. Drug-Induced Extrapyramidal Syndromes: Implications for Contemporary Practice. Psychiatr. Clin. North Am. 2016; 39: 391–411. DOI: 10.1016/j.psc.2016.04.003 27514296

[4] Carbon M, Hsieh C-H, Kane JM, Correll CU. Tardive Dyskinesia Prevalence in the Period of Second-Generation Antipsychotic Use: A Meta-Analysis. J. Clin. Psychiatry. 2017; 78; e264–e278. DOI: 10.4088/JCP.16r10832 28146614

[5] Correll CU, Leucht S, Kane JM. Lower risk for tardive dyskinesia associated with second-generation antipsychotics: a systematic review of 1-year studies. Am. J. Psychiatry. 2004; 161: 414–425. DOI: 10.1176/appi.ajp.161.3.414 14992963

[6] Chouinard G, Chouinard V-A. Atypical antipsychotics: CATIE study, drug-induced movement disorder and resulting iatrogenic psychiatric-like symptoms, supersensitivity rebound psychosis and withdrawal discontinuation syndromes. Psychother. Psychosom. 2008; 77: 69–77. DOI: 10.1159/000112883 18230939

[7] Maeda K, et al. Brexpiprazole I: in vitro and in vivo characterization of a novel serotonin-dopamine activity modulator. J. Pharmacol. Exp. Ther. 2014; 350: 589–604. DOI: 10.1124/jpet.114.213793 24947465

[8] Obara K, et al. Inhibition of Recombinant Human Acetylcholinesterase Activity by Antipsychotics. Pharmacology. 2019; 104: 43–50. DOI: 10.1159/000500227 31067549

[9] Correll CU, et al. Efficacy and Safety of Brexpiprazole for the Treatment of Acute Schizophrenia: A 6-Week Randomized, Double-Blind, Placebo-Controlled Trial. Am. J. Psychiatry. 2015; 172: 870–880. DOI: 10.1176/appi.ajp.2015.14101275 25882325

[10] Kane JM, et al. A multicenter, randomized, double-blind, controlled phase 3 trial of fixed-dose brexpiprazole for the treatment of adults with acute schizophrenia. Schizophr. Res. 2015; 164: 127–135. DOI: 10.1016/j.schres.2015.01.038 25682550

[11] Ishigooka J, Iwashita S, Tadori Y. Long-term safety and effectiveness of brexpiprazole in Japanese patients with schizophrenia: A 52-week, open-label study. Psychiatry Clin. Neurosci. 2018; 72: 445–453. DOI: 10.1111/pcn.12654 29582518

[12] Thase ME, et al. Efficacy and Safety of Adjunctive Brexpiprazole 2 mg in Major Depressive Disorder: A Phase 3, Randomized, Placebo-Controlled Study in Patients With Inadequate Response to Antidepressants. J. Clin. Psychiatry. 2015; 76: 6108. DOI: 10.4088/JCP.14m09688 26301701

[13] Ishigooka J, Iwashita S, Tadori Y. Efficacy and safety of brexpiprazole for the treatment of acute schizophrenia in Japan: A 6-week, randomized, double-blind, placebo-controlled study. Psychiatry Clin. Neurosci. 2018; 72: 692–700. DOI: 10.1111/pcn.12682 29774628

[14] Fleischhacker WW, et al. Efficacy and Safety of Brexpiprazole (OPC-34712) as Maintenance Treatment in Adults with Schizophrenia: a Randomized, Double-Blind, Placebo-Controlled Study. Int. J. Neuropsychopharmacol. 2016; 20: 11–21. DOI: 10.1093/ijnp/pyw076 PMC5412583 27566723

[15] Kishi T, et al. Comparison of the efficacy and safety of 4 and 2 mg/day brexpiprazole for acute schizophrenia: a meta-analysis of double-blind, randomized placebo-controlled trials. Neuropsychiatr. Dis. Treat. 2018; 14: 2519–2530. DOI: 10.2147/NDT.S176676 30319261 PMC6171755

[16] Thase ME, et al. Adjunctive brexpiprazole 1 and 3 mg for patients with major depressive disorder following inadequate response to antidepressants: a phase 3, randomized, double-blind study. J. Clin. Psychiatry. 2015; 76: 1232–1240. DOI: 10.4088/JCP.14m09689 26301771

[17] Malla A, et al. The effect of brexpiprazole in adult outpatients with early-episode schizophrenia: an exploratory study. Int. Clin. Psychopharmacol. 2016; 31: 307–314. DOI: 10.1097/YIC.0000000000000140 27571460 PMC5049948

[18] Forbes A, et al. A Long-Term, Open-Label Study to Evaluate the Safety and Tolerability of Brexpiprazole as Maintenance Treatment in Adults with Schizophrenia. Int. J. Neuropsychopharmacol. 2018; 21: 433–441. DOI: 10.1093/ijnp/pyy002 29415258 PMC5932477

[19] Sharma VD, Gupta HV, Espay AJ. Copulatory Dyskinesia: Pathognomonic Manifestation of Tardive Dyskinesia. Tremor Hyperkinetic Mov. 10: 56. DOI: 10.5334/tohm.585 PMC7747756 33362950

[20] Jackowiak EM, Chou KL. Severe parkinsonism caused by brexpiprazole: A case report. Parkinsonism Relat. Disord. 2019; 69: 138–139. DOI: 10.1016/j.parkreldis.2019.11.013 31756572

[21] Sanagawa A, Shiraishi N, Sekiguchi F, Akechi T, Kimura K. Successful Use of Brexpiprazole for Parkinson’s Disease Psychosis Without Adverse Effects: A Case Report. J. Clin. Psychopharmacol. 2019; 39: 685–687. DOI: 10.1097/JCP.0000000000001127 31688389

[22] Citrome L, et al. The effect of brexpiprazole (OPC-34712) and aripiprazole in adult patients with acute schizophrenia: results from a randomized, exploratory study. Int. Clin. Psychopharmacol. 2016; 31: 192–201. DOI: 10.1097/YIC.0000000000000123 26963842

[23] Correll CU, et al. Successful switching of patients with acute schizophrenia from another antipsychotic to brexpiprazole: comparison of clinicians’ choice of cross-titration schedules in a post hoc analysis of a randomized, double-blind, maintenance treatment study. CNS Spectr. 2019; 24: 507–517. DOI: 10.1017/S1092852918001086 30306884

[24] Kishi T, Ikuta T, Matsuda Y, Sakuma K, Iwata N. Aripiprazole vs. brexpiprazole for acute schizophrenia: a systematic review and network meta-analysis. Psychopharmacology (Berl.). 2020; 237: 1459–1470. DOI: 10.1007/s00213-020-05472-5 32002559

[25] Citrome L. Activating and Sedating Adverse Effects of Second-Generation Antipsychotics in the Treatment of Schizophrenia and Major Depressive Disorder: Absolute Risk Increase and Number Needed to Harm. J. Clin. Psychopharmacol. 2017; 37: 138–147. DOI: 10.1097/JCP.0000000000000665 28141623

[26] Citrome L. The ABC’s of dopamine receptor partial agonists – aripiprazole, brexpiprazole and cariprazine: the 15-min challenge to sort these agents out. Int. J. Clin. Pract. 2015; 69: 1211–1220. DOI: 10.1111/ijcp.12752 26477545

[27] Misiak B, Bieńkowski P, Samochowiec J. Cariprazine – a novel antipsychotic drug and its place in the treatment of schizophrenia. Psychiatr. Pol. 2018; 52: 971–981. DOI: 10.12740/PP/OnlineFirst/80710 30659560

[28] Carnicella S, et al. Implication of dopamine D3 receptor activation in the reversion of Parkinson’s disease-related motivational deficits. Transl. Psychiatry. 2014; 4: e401–e401. DOI: 10.1038/tp.2014.43 24937095 PMC4080324

[29] Citrome L. Cariprazine: chemistry, pharmacodynamics, pharmacokinetics, and metabolism, clinical efficacy, safety, and tolerability. Expert Opin. Drug Metab. Toxicol. 2013; 9: 193–206. DOI: 10.1517/17425255.2013.759211 23320989

[30] Durgam S, et al. Cariprazine in the treatment of schizophrenia: a proof-of-concept trial. Int. Clin. Psychopharmacol. 2016; 31: 61–68. DOI: 10.1097/YIC.0000000000000110 26655732 PMC4736298

[31] Durgam S, et al. An evaluation of the safety and efficacy of cariprazine in patients with acute exacerbation of schizophrenia: a phase II, randomized clinical trial. Schizophr. Res. 2014; 152: 450–457. DOI: 10.1016/j.schres.2013.11.041 24412468

[32] Kane JM, et al. Efficacy and Safety of Cariprazine in Acute Exacerbation of Schizophrenia: Results From an International, Phase III Clinical Trial. J. Clin. Psychopharmacol. 2015; 35: 367–373. DOI: 10.1097/JCP.0000000000000346 26075487

[33] Németh G, et al. Cariprazine versus risperidone monotherapy for treatment of predominant negative symptoms in patients with schizophrenia: a randomised, double-blind, controlled trial. The Lancet. 2017; 389: 1103–1113. DOI: 10.1016/S0140-6736(17)30060-0 28185672

[34] Ivanov SV, et al. Early Clinical Effects of Novel Partial D3/D2 Agonist Cariprazine in Schizophrenia Patients With Predominantly Negative Symptoms (Open-Label, Non-controlled Study). Front. Psychiatry. 2022; 12: 770592. DOI: 10.3389/fpsyt.2021.770592 35140638 PMC8818881

[35] Durgam S, et al. Cariprazine in Acute Exacerbation of Schizophrenia: A Fixed-Dose, Phase 3, Randomized, Double-Blind, Placebo- and Active-Controlled Trial. J. Clin. Psychiatry. 2015; 76: 2310. DOI: 10.4088/JCP.15m09997 26717533

[36] Nasrallah HA, et al. The safety and tolerability of cariprazine in long-term treatment of schizophrenia: a post hoc pooled analysis. BMC Psychiatry. 2017; 17: 305. DOI: 10.1186/s12888-017-1459-z 28836957 PMC5571492

[37] Kane JM, et al. Efficacy and Safety of Cariprazine in Acute Exacerbation of Schizophrenia: Results From an International, Phase III Clinical Trial. J. Clin. Psychopharmacol. 2015; 35: 367–373. DOI: 10.1097/JCP.0000000000000346 26075487

[38] Earley W, et al. Safety and tolerability of cariprazine in patients with acute exacerbation of schizophrenia: a pooled analysis of four phase II/III randomized, double-blind, placebo-controlled studies. Int. Clin. Psychopharmacol. 2017; 32: 319–328. DOI: 10.1097/YIC.0000000000000187 28692485 PMC5625952

[39] Barabássy Á, et al. Safety and Tolerability of Cariprazine in Patients with Schizophrenia: A Pooled Analysis of Eight Phase II/III Studies. Neuropsychiatr. Dis. Treat. 2021; 17: 957–970. DOI: 10.2147/NDT.S301225 33854317 PMC8040316

[40] Szatmári B, et al. Cariprazine Safety in Adolescents and the Elderly: Analyses of Clinical Study Data. Front. Psychiatry. 2020; 11: 61. DOI: 10.3389/fpsyt.2020.00061 32194443 PMC7062963

[41] Durgam S, et al. Efficacy and Safety of Adjunctive Cariprazine in Inadequate Responders to Antidepressants: A Randomized, Double-Blind, Placebo-Controlled Study in Adult Patients With Major Depressive Disorder. J. Clin. Psychiatry. 2016; 77: 6112. DOI: 10.4088/JCP.15m10070 27046309

[42] Durgam S, et al. The efficacy and tolerability of cariprazine in acute mania associated with bipolar I disorder: a phase II trial. Bipolar Disord. 2015; 17: 63–75. DOI: 10.1111/bdi.12238 25056368

[43] Ketter TA, et al. The safety and tolerability of cariprazine in patients with manic or mixed episodes associated with bipolar I disorder: A 16-week open-label study. J. Affect. Disord. 2018; 225: 350–356. DOI: 10.1016/j.jad.2017.08.040 28843918

[44] Yatham LN, Vieta E, Earley W. Evaluation of cariprazine in the treatment of bipolar I and II depression: a randomized, double-blind, placebo-controlled, phase 2 trial. Int. Clin. Psychopharmacol. 2020; 35: 147–156. DOI: 10.1097/YIC.0000000000000307 32058426 PMC7099842

[45] Earley W, et al. Tolerability of cariprazine in the treatment of acute bipolar I mania: A pooled post hoc analysis of 3 phase II/III studies. J. Affect. Disord. 2017; 215: 205–212. DOI: 10.1016/j.jad.2017.03.032 28343051

[46] Citrome L, et al. Cariprazine and akathisia, restlessness, and extrapyramidal symptoms in patients with bipolar depression. J. Affect. Disord. 2021; 288: 191–198. DOI: 10.1016/j.jad.2021.03.076 33915374

[47] Lao KSJ, He Y, Wong ICK, Besag FMC, Chan EW. Tolerability and Safety Profile of Cariprazine in Treating Psychotic Disorders, Bipolar Disorder and Major Depressive Disorder: A Systematic Review with Meta-Analysis of Randomized Controlled Trials. CNS Drugs. 2016; 30: 1043–1054. DOI: 10.1007/s40263-016-0382-z 27550371

[48] Kadakia A, et al. Efficacy and tolerability of atypical antipsychotics for acute bipolar depression: a network meta-analysis. BMC Psychiatry. 2021; 21: 249. DOI: 10.1186/s12888-021-03220-3 33975574 PMC8112003

[49] Blair HA. Lumateperone: First Approval. Drugs. 2020; 80: 417–423. DOI: 10.1007/s40265-020-01271-6 32060882

[50] Edinoff A, et al. Lumateperone for the Treatment of Schizophrenia. Psychopharmacol. Bull. 2020; 50: 32–59. 33012872 10.64719/pb.4372 PMC7511146

[51] Lieberman JA, et al. ITI-007 for the Treatment of Schizophrenia: A 4-Week Randomized, Double-Blind, Controlled Trial. Biol. Psychiatry. 2016; 79: 952–961. DOI: 10.1016/j.biopsych.2015.08.026 26444072

[52] Correll CU, et al. Efficacy and Safety of Lumateperone for Treatment of Schizophrenia: A Randomized Clinical Trial. JAMA Psychiatry. 2020; 77: 349–358. DOI: 10.1001/jamapsychiatry.2019.4379 31913424 PMC6990963

[53] Vanover K, et al. 30 Lumateperone (ITI-007) for the Treatment of Schizophrenia: Overview of Placebo-Controlled Clinical Trials and an Open-label Safety Switching Study. CNS Spectr. 2019; 24: 190–191. DOI: 10.1017/S1092852919000245

[54] Kane JM, et al. Safety and tolerability of lumateperone for the treatment of schizophrenia: a pooled analysis of late-phase placebo- and active-controlled clinical trials. Int. Clin. Psychopharmacol. 2021; 36: 244–250. DOI: 10.1097/YIC.0000000000000371 34054112 PMC8322041

[55] Calabrese JR, et al. Efficacy and Safety of Lumateperone for Major Depressive Episodes Associated With Bipolar I or Bipolar II Disorder: A Phase 3 Randomized Placebo-Controlled Trial. Am. J. Psychiatry. 2021; 178: 1098–1106. DOI: 10.1176/appi.ajp.2021.20091339 34551584

[56] Corponi F, et al. Novel antipsychotics specificity profile: A clinically oriented review of lurasidone, brexpiprazole, cariprazine and lumateperone. Eur. Neuropsychopharmacol. 2019; 29: 971–985. DOI: 10.1016/j.euroneuro.2019.06.008 31255396

[57] Ogasa M, Kimura T, Nakamura M, Guarino J. Lurasidone in the treatment of schizophrenia: a 6-week, placebo-controlled study. Psychopharmacology (Berl.). 2013; 225: 519–530. DOI: 10.1007/s00213-012-2838-2 22903391 PMC3546299

[58] Nasrallah HA, et al. Lurasidone for the treatment of acutely psychotic patients with schizophrenia: A 6-week, randomized, placebo-controlled study. J. Psychiatr. Res. 2013; 47: 670–677. DOI: 10.1016/j.jpsychires.2013.01.020 23421963

[59] Stahl SM, et al. Effectiveness of Lurasidone for Patients With Schizophrenia Following 6 Weeks of Acute Treatment With Lurasidone, Olanzapine, or Placebo: A 6-Month, Open-Label, Extension Study. J. Clin. Psychiatry. 2013; 74: 5562. DOI: 10.4088/JCP.12m08084 23541189

[60] Tandon R, et al. A double-blind, placebo-controlled, randomized withdrawal study of lurasidone for the maintenance of efficacy in patients with schizophrenia. J. Psychopharmacol. (Oxf.). 2016; 30: 69–77. DOI: 10.1177/0269881115620460 PMC4717319 26645209

[61] Loebel A, et al. Lurasidone Monotherapy in the Treatment of Bipolar I Depression: A Randomized, Double-Blind, Placebo-Controlled Study. Am. J. Psychiatry. 2014; 171: 160–168. DOI: 10.1176/appi.ajp.2013.13070984 24170180

[62] Loebel A, et al. Lurasidone as Adjunctive Therapy With Lithium or Valproate for the Treatment of Bipolar I Depression: A Randomized, Double-Blind, Placebo-Controlled Study. Am. J. Psychiatry. 2014; 171: 169–177. DOI: 10.1176/appi.ajp.2013.13070985 24170221

[63] Suppes T, Kroger H, Pikalov A, Loebel A. Lurasidone adjunctive with lithium or valproate for bipolar depression: A placebo-controlled trial utilizing prospective and retrospective enrolment cohorts. J. Psychiatr. Res. 2016; 78: 86–93. DOI: 10.1016/j.jpsychires.2016.03.012 27089521

[64] Suppes T, et al. Lurasidone for the Treatment of Major Depressive Disorder With Mixed Features: A Randomized, Double-Blind, Placebo-Controlled Study. Am. J. Psychiatry. 2016; 173: 400–407. DOI: 10.1176/appi.ajp.2015.15060770 26552942

[65] Higuchi T, et al. Lurasidone in the long-term treatment of Japanese patients with bipolar I disorder: a 52 week open label study. Int. J. Bipolar Disord. 2021; 9: 25. DOI: 10.1186/s40345-021-00230-8 34342746 PMC8333182

[66] Citrome L, et al. Long-term safety and tolerability of lurasidone in schizophrenia: a 12-month, double-blind, active-controlled study. Int. Clin. Psychopharmacol. 2012; 27: 165–176. DOI: 10.1097/YIC.0b013e32835281ef 22395527

[67] Citrome L, et al. Effectiveness of lurasidone in schizophrenia or schizoaffective patients switched from other antipsychotics: a 6-month, open-label, extension study. CNS Spectr. 2014; 19: 330–339. DOI: 10.1017/S109285291300093X 24330868 PMC4140225

[68] Kishi T, Nosaka T, Sakuma K, Okuya M, Iwata N. Efficacy, tolerability, and safety of lurasidone for acute schizophrenia: A systematic review and network meta-analysis of phase 3 trials in Japan. Neuropsychopharmacol. Rep. 2020; 40: 314–322. DOI: 10.1002/npr2.12131 32767739 PMC7722667

[69] Ketter TA, et al. Lurasidone in the Long-Term Treatment of Patients with Bipolar Disorder: A 24-Week Open-Label Extension Study. Depress. Anxiety. 2016; 33: 424–434. DOI: 10.1002/da.22479 26918425 PMC5069590

[70] Ishigooka J, et al. Lurasidone in the Long-Term Treatment of Bipolar I Depression: A 28-week Open Label Extension Study. J. Affect. Disord. 2021; 281: 160–167. DOI: 10.1016/j.jad.2020.12.005 33321381

[71] Calabrese JR, et al. Lurasidone in combination with lithium or valproate for the maintenance treatment of bipolar I disorder. Eur. Neuropsychopharmacol. 2017; 27: 865–876. DOI: 10.1016/j.euroneuro.2017.06.013 28689688

[72] Tripathi R, Reich SG, Scorr L, Guardiani E, Factor SA. Lurasidone-Induced Tardive Syndrome. Mov. Disord. Clin. Pract. 2019; 6: 601–604. DOI: 10.1002/mdc3.12812 31538095 PMC6749798

[73] Schultz J, Furnish K, El-Mallakh RS. Consumption of Aspartame Associated with Tardive Dyskinesia: New Observation. J. Clin. Psychopharmacol. 2019; 39: 690–691. DOI: 10.1097/JCP.0000000000001112 31688394

[74] Reichenberg JL, Ridout KK, Rickler KC. Lurasidone – Induced Rabbit Syndrome: A Case Report. J. Clin. Psychiatry. 2017; 78: e553. DOI: 10.4088/JCP.16cr11157 28570801 PMC6563912

[75] Caffrey D, Sowden GL. A missed case of lurasidone induced laryngospasm: A case study and overview of extrapyramidal symptom identification and treatment. Int. J. Psychiatry Med. 2021; 56: 73–82. DOI: 10.1177/0091217420943786 32660283

[76] Suthar N, Aneja J. Lurasidone-induced Parkinsonism and Hyperprolactinemia. Indian J. Psychol. Med. 2019; 41: 192–194. DOI: 10.4103/IJPSYM.IJPSYM_274_18 30983673 PMC6436398

[77] Meltzer HY, et al. Lurasidone in the Treatment of Schizophrenia: A Randomized, Double-Blind, Placebo- and Olanzapine-Controlled Study. Am. J. Psychiatry. 2011; 168: 957–967. DOI: 10.1176/appi.ajp.2011.10060907 21676992

[78] Loebel A, et al. Efficacy and safety of lurasidone 80mg/day and 160mg/day in the treatment of schizophrenia: A randomized, double-blind, placebo- and active-controlled trial. Schizophr. Res. 2013; 145: 101–109. DOI: 10.1016/j.schres.2013.01.009 23415311

[79] Loebel A, et al. Effectiveness of lurasidone vs. quetiapine XR for relapse prevention in schizophrenia: A 12-month, double-blind, noninferiority study. Schizophr. Res. 2013; 147: 95–102. DOI: 10.1016/j.schres.2013.03.013 23583011

[80] Kishi T, Yoshimura R, Sakuma K, Okuya M, Iwata N. Lurasidone, olanzapine, and quetiapine extended-release for bipolar depression: A systematic review and network meta-analysis of phase 3 trials in Japan. Neuropsychopharmacol. Rep. 2020; 40: 417–422. DOI: 10.1002/npr2.12137 32902200 PMC7722645

[81] Potkin SG, Ogasa M, Cucchiaro J, Loebel A. Double-blind comparison of the safety and efficacy of lurasidone and ziprasidone in clinically stable outpatients with schizophrenia or schizoaffective disorder. Schizophr. Res. 2011; 132: 101–107. DOI: 10.1016/j.schres.2011.04.008 21889878

[82] Leucht S, et al. Comparative efficacy and tolerability of 15 antipsychotic drugs in schizophrenia: a multiple-treatments meta-analysis. The Lancet. 2013; 382: 951–962. DOI: 10.1016/S0140-6736(13)60733-3 23810019

[83] Ostacher M, et al. Lurasidone compared to other atypical antipsychotic monotherapies for bipolar depression: A systematic review and network meta-analysis. World J. Biol. Psychiatry Off. J. World Fed. Soc. Biol. Psychiatry. 2018; 19: 586–601. DOI: 10.1080/15622975.2017.1285050 28264635

[84] Meltzer HY, et al. Pimavanserin, a selective serotonin (5-HT)2A-inverse agonist, enhances the efficacy and safety of risperidone, 2mg/day, but does not enhance efficacy of haloperidol, 2mg/day: Comparison with reference dose risperidone, 6mg/day. Schizophr. Res. 2012; 141: 144–152. DOI: 10.1016/j.schres.2012.07.029 22954754

[85] Vanover KE, et al. Pharmacological and Behavioral Profile of N-(4-Fluorophenylmethyl)-N-(1-methylpiperidin-4-yl)-N′-(4-(2-methylpropyloxy)phenylmethyl) Carbamide (2R,3R)-Dihydroxybutanedioate (2:1) (ACP-103), a Novel 5-Hydroxytryptamine2A Receptor Inverse Agonist. J. Pharmacol. Exp. Ther. 2006; 317: 910–918. DOI: 10.1124/jpet.105.097006 16469866

[86] Meltzer HY, et al. Pimavanserin, a Serotonin2A Receptor Inverse Agonist, for the Treatment of Parkinson’s Disease Psychosis. Neuropsychopharmacology. 2010; 35: 881–892. DOI: 10.1038/npp.2009.176 19907417 PMC3055369

[87] Cummings J, et al. Pimavanserin for patients with Parkinson’s disease psychosis: a randomised, placebo-controlled phase 3 trial. The Lancet. 2014; 383: 533–540. DOI: 10.1016/S0140-6736(13)62106-6 24183563

[88] Bugarski-Kirola D, et al. Pimavanserin for negative symptoms of schizophrenia: results from the ADVANCE phase 2 randomised, placebo-controlled trial in North America and Europe. Lancet Psychiatry. 2022; 9: 46–58. DOI: 10.1016/S2215-0366(21)00386-2 34861170

[89] ACADIA Pharmaceuticals Inc. A Phase 3, Randomized, Double-Blind, Placebo-Controlled Study to Evaluate the Efficacy and Safety of Adjunctive Pimavanserin for the Treatment of Schizophrenia; 2020. https://clinicaltrials.gov/ct2/show/results/NCT02970292

[90] ACADIA Pharmaceuticals Inc. A Phase 2, Randomized, Double-Blind, Placebo-Controlled Study to Evaluate the Efficacy and Safety of Pimavanserin as Adjunctive Treatment for the Negative Symptoms of Schizophrenia; 2020. https://clinicaltrials.gov/ct2/show/results/NCT02970305 10.1093/schbul/sbaf034 PMC12809779 40181715

[91] Lobo MC, Whitehurst TS, Kaar SJ, Howes OD. New and emerging treatments for schizophrenia: a narrative review of their pharmacology, efficacy and side effect profile relative to established antipsychotics. Neurosci. Biobehav. Rev. 2022; 132: 324–361. DOI: 10.1016/j.neubiorev.2021.11.032 34838528 PMC7616977

[92] Monahan C, McCoy L, Powell J, Gums JG. Olanzapine/Samidorphan: New Drug Approved for Treating Bipolar I Disorder and Schizophrenia. Ann. Pharmacother; 2022. DOI: 10.1177/10600280211070330 35040357

[93] Potkin SG, et al. Efficacy and Safety of a Combination of Olanzapine and Samidorphan in Adult Patients With an Acute Exacerbation of Schizophrenia: Outcomes From the Randomized, Phase 3 ENLIGHTEN-1 Study. J. Clin. Psychiatry. 2020; 81: 5960. DOI: 10.4088/JCP.19m12769 32141723

[94] Correll CU, et al. Effects of Olanzapine Combined With Samidorphan on Weight Gain in Schizophrenia: A 24-Week Phase 3 Study. Am. J. Psychiatry. 2020; 177: 1168–1178. DOI: 10.1176/appi.ajp.2020.19121279 32791894

[95] Sun L, McDonnell D, von Moltke L. Pharmacokinetics and Short-term Safety of ALKS 3831, a Fixed-dose Combination of Olanzapine and Samidorphan, in Adult Subjects with Schizophrenia. Clin. Ther. 2018; 40: 1845–1854.e2. DOI: 10.1016/j.clinthera.2018.09.002 30348514

[96] Martin WF, et al. Mitigation of Olanzapine-Induced Weight Gain With Samidorphan, an Opioid Antagonist: A Randomized Double-Blind Phase 2 Study in Patients With Schizophrenia. Am. J. Psychiatry. 2019; 176: 457–467. DOI: 10.1176/appi.ajp.2018.18030280 30845818

[97] Brunette MF, et al. Olanzapine Plus Samidorphan (ALKS 3831) in Schizophrenia and Comorbid Alcohol Use Disorder: A Phase 2, Randomized Clinical Trial. J. Clin. Psychiatry. 2020; 81: 13176. DOI: 10.4088/JCP.19m12786 32160422

[98] Yagoda S, et al. Long-term safety and durability of effect with a combination of olanzapine and samidorphan in patients with schizophrenia: results from a 1-year open-label extension study. CNS Spectr. 2021; 26: 383–392. DOI: 10.1017/S1092852920001376 32393412

[99] Valenstein M, et al. Poor Antipsychotic Adherence Among Patients With Schizophrenia: Medication and Patient Factors. Schizophr. Bull. 2004; 30: 255–264. DOI: 10.1093/oxfordjournals.schbul.a007076 15279044

[100] Park S-C, et al. Comparative efficacy and safety of long-Acting injectable and oral second-generation antipsychotics for the treatment of schizophrenia: A systematic review and meta-Analysis. Clin. Psychopharmacol. Neurosci. 2018; 16: 361–375. DOI: 10.9758/cpn.2018.16.4.361 30466208 PMC6245299

[101] Leucht C, et al. Oral versus depot antipsychotic drugs for schizophrenia—A critical systematic review and meta-analysis of randomised long-term trials. Schizophr. Res. 2011; 127: 83–92. DOI: 10.1016/j.schres.2010.11.020 21257294

[102] Saucedo Uribe E, et al. Preliminary efficacy and tolerability profiles of first versus second-generation Long-Acting Injectable Antipsychotics in schizophrenia: A systematic review and meta-analysis. J. Psychiatr. Res. 2020; 129: 222–233. DOI: 10.1016/j.jpsychires.2020.06.013 32805530

[103] Filts Y, et al. Long-term efficacy and safety of once-monthly Risperidone ISM® in the treatment of schizophrenia: Results from a 12-month open-label extension study. Schizophr. Res. 2022; 239: 83–91. DOI: 10.1016/j.schres.2021.11.030 34847501

[104] Eerdekens M, Rasmussen M, Vermeulen A, Lowenthal R, Peer AV. 102. Kinetics and safety of a novel risperidone depot formulation. Biol. Psychiatry. 2000; 47: S31. DOI: 10.1016/S0006-3223(00)00364-4

[105] Jarema M, Bieńkowski P, Heitzman J, Parnowski T, Rybakowski J. Paliperidone palmitate: effectiveness, safety, and the use for treatment of schizophrenia. Psychiatr. Pol. 2017; 51: 7–21. DOI: 10.12740/PP/64581 28455891

[106] Bobo WV, Shelton RC. Risperidone long-acting injectable (Risperdal Consta®) for maintenance treatment in patients with bipolar disorder. Expert Rev. Neurother. 2010; 10: 1637–1658. DOI: 10.1586/ern.10.143 20977322

[107] Carabias LA, et al. A phase II study to evaluate the pharmacokinetics, safety, and tolerability of Risperidone ISM multiple intramuscular injections once every 4 weeks in patients with schizophrenia. Int. Clin. Psychopharmacol. 2018; 33: 79–87. DOI: 10.1097/YIC.0000000000000203 29112001

[108] Malempati RN, et al. Long-term efficacy of risperidone long-acting injectable in bipolar disorder with psychotic features: a prospective study of 3-year outcomes. Int. Clin. Psychopharmacol. 2011; 26: 146–150. DOI: 10.1097/YIC.0b013e328343ba60 21372721

[109] Kimura H, et al. Risperidone long-acting injectable in the treatment of treatment-resistant schizophrenia with dopamine supersensitivity psychosis: Results of a 2-year prospective study, including an additional 1-year follow-up. J. Psychopharmacol. (Oxf.). 2016; 30: 795–802. DOI: 10.1177/0269881116655978 27371496

[110] Gharabawi GM, Bossie CA, Zhu Y, Mao L, Lasser RA. An assessment of emergent tardive dyskinesia and existing dyskinesia in patients receiving long-acting, injectable risperidone: results from a long-term study. Schizophr. Res. 2005; 77: 129–139. DOI: 10.1016/j.schres.2005.03.015 15913962

[111] Chung AKK. Atypical presentation of tardive dyskinesia associated with risperidone long-acting injection as maintenance treatment in bipolar affective disorder: a case report. Curr. Drug Saf. 2012; 7: 21–23. DOI: 10.2174/157488612800492807 22663952

[112] Ostuzzi G, Bighelli I, So R, Furukawa TA, Barbui C. Does formulation matter? A systematic review and meta-analysis of oral versus long-acting antipsychotic studies. Schizophr. Res. 2017; 183: 10–21. DOI: 10.1016/j.schres.2016.11.010 27866695

[113] Sampson S, Hosalli P, Furtado VA, Davis JM. Risperidone (depot) for schizophrenia. Cochrane Database Syst. Rev. 2016; 4: CD004161. DOI: 10.1002/14651858.CD004161.pub2 27078222 PMC10493848

[114] Nasrallah HA, et al. A Controlled, Evidence-Based Trial of Paliperidone Palmitate, A Long-Acting Injectable Antipsychotic, in Schizophrenia. Neuropsychopharmacology. 2010; 35: 2072–2082. DOI: 10.1038/npp.2010.79 20555312 PMC3055301

[115] Kramer M, et al. Paliperidone palmitate, a potential long-acting treatment for patients with schizophrenia. Results of a randomized, double-blind, placebo-controlled efficacy and safety study. Int. J. Neuropsychopharmacol. 2010; 13: 635–647. DOI: 10.1017/S1461145709990988 19941696

[116] Berwaerts J, et al. Efficacy and Safety of the 3-Month Formulation of Paliperidone Palmitate vs Placebo for Relapse Prevention of Schizophrenia: A Randomized Clinical Trial. JAMA Psychiatry. 2015; 72: 830–839. DOI: 10.1001/jamapsychiatry.2015.0241 25820612

[117] Emsley R, Kilian S. Efficacy and safety profile of paliperidone palmitate injections in the management of patients with schizophrenia: an evidence-based review. Neuropsychiatr. Dis. Treat. 2018; 14: 205–223. DOI: 10.2147/NDT.S139633 29379293 PMC5759847

[118] Zhao J, et al. Safety and efficacy of paliperidone palmitate 1-month formulation in Chinese patients with schizophrenia: a 25-week, open-label, multicenter, Phase IV study. Neuropsychiatr. Dis. Treat. 2017; 13: 2045–2056. DOI: 10.2147/NDT.S131224 28814873 PMC5546821

[119] Gopal S, et al. Incidence and time course of extrapyramidal symptoms with oral and long-acting injectable paliperidone: a posthoc pooled analysis of seven randomized controlled studies. Neuropsychiatr. Dis. Treat. 2013; 9: 1381–1392. DOI: 10.2147/NDT.S49944 24092977 PMC3788701

[120] Gopal S, et al. Incidence of tardive dyskinesia: a comparison of long-acting injectable and oral paliperidone clinical trial databases. Int. J. Clin. Pract. 2014; 68: 1514–1522. DOI: 10.1111/ijcp.12493 25358867 PMC4265240

[121] Takada R, Yamamuro K, Kishimoto T. Long-lasting extrapyramidal symptoms after multiple injections of paliperidone palmitate to treat schizophrenia. Neuropsychiatr. Dis. Treat. 2018; 14: 2541–2544. DOI: 10.2147/NDT.S176478 30323602 PMC6174309

[122] Ma C-H, Chien Y-L, Liu C-C, Chen I-M, Lin C-H. A case of tardive dystonia associated with long-acting injectable paliperidone palmitate. Eur. Neuropsychopharmacol. 2016; 26: 1251–1252. DOI: 10.1016/j.euroneuro.2016.04.006 27130697

[123] Lally J, Byrne F, Walsh E. A case of paliperidone-palmitate-induced tardive dyskinesia. Gen. Hosp. Psychiatry. 2013; 35: 213.e5–7. DOI: 10.1016/j.genhosppsych.2012.04.009 22703608

[124] Jang S, Woo J. Five Month-Persistent Extrapyramidal Symptoms following a Single Injection of Paliperidone Palmitate: A Case Report. Clin. Psychopharmacol. Neurosci. Off. Sci. J. Korean Coll. Neuropsychopharmacol. 2017; 15: 288–291. DOI: 10.9758/cpn.2017.15.3.288 PMC5565081 28783941

[125] Li H, Rui Q, Ning X, Xu H, Gu N. A comparative study of paliperidone palmitate and risperidone long-acting injectable therapy in schizophrenia. Prog. Neuropsychopharmacol. Biol. Psychiatry. 2011; 35: 1002–1008. DOI: 10.1016/j.pnpbp.2011.02.001 21315787

[126] Pandina G, et al. A double-blind study of paliperidone palmitate and risperidone long-acting injectable in adults with schizophrenia. Prog. Neuropsychopharmacol. Biol. Psychiatry. 2011; 35: 218–226. DOI: 10.1016/j.pnpbp.2010.11.008 21092748

[127] Nussbaum AM, Stroup TS. Paliperidone palmitate for schizophrenia. Cochrane Database Syst. Rev; 2012; CD008296. DOI: 10.1002/14651858.CD008296.pub2 22696377 PMC12145955

[128] Saglam Aykut D. Comparison of Paliperidone Palmitate and Second-Generation Oral Antipsychotics in Terms of Medication Adherence, Side Effects, and Quality of Life. J. Clin. Psychopharmacol. 2019; 39: 57–62. DOI: 10.1097/JCP.0000000000000993 30566417

[129] Kim E, Correll CU, Mao L, Starr HL, Alphs L. Once-monthly paliperidone palmitate compared with conventional and atypical daily oral antipsychotic treatment in patients with schizophrenia. CNS Spectr. 2016; 21: 466–477. DOI: 10.1017/S1092852916000444 27629292

[130] Naber D, et al. Qualify: a randomized head-to-head study of aripiprazole once-monthly and paliperidone palmitate in the treatment of schizophrenia. Schizophr. Res. 2015; 168: 498–504. DOI: 10.1016/j.schres.2015.07.007 26232241

[131] Citrome L. Aripiprazole long-acting injectable formulations for schizophrenia: aripiprazole monohydrate and aripiprazole lauroxil. Expert Rev. Clin. Pharmacol. 2016; 9: 169–186. DOI: 10.1586/17512433.2016.1121809 26573020

[132] Maini K, et al. Aripiprazole Lauroxil, a Novel Injectable Long-Acting Antipsychotic Treatment for Adults with Schizophrenia: A Comprehensive Review. Neurol. Int. 2021; 13: 279–296. DOI: 10.3390/neurolint13030029 34287335 PMC8293312

[133] Allen JA, et al. Discovery of β-arrestin-biased dopamine D2 ligands for probing signal transduction pathways essential for antipsychotic efficacy. Proc. Natl. Acad. Sci. U. S. A. 2011; 108: 18488–18493. DOI: 10.1073/pnas.1104807108 22025698 PMC3215024

[134] Kane JM, et al. Aripiprazole Once-Monthly in the Acute Treatment of Schizophrenia: Findings From a 12-Week, Randomized, Double-Blind, Placebo-Controlled Study. J. Clin. Psychiatry. 2014; 75: 1347. DOI: 10.4088/JCP.14m09168 25188501

[135] Meltzer HY, et al. A Randomized, Double-Blind, Placebo-Controlled Trial of Aripiprazole Lauroxil in Acute Exacerbation of Schizophrenia. J. Clin. Psychiatry. 2015; 76: 16764. DOI: 10.4088/JCP.14m09741 26114240

[136] Kane JM, et al. Aripiprazole intramuscular depot as maintenance treatment in patients with schizophrenia: a 52-week, multicenter, randomized, double-blind, placebo-controlled study. J. Clin. Psychiatry. 2012; 73: 617–624. DOI: 10.4088/JCP.11m07530 22697189

[137] Citrome L. Asenapine review, part I: chemistry, receptor affinity profile, pharmacokinetics and metabolism. Expert Opin. Drug Metab. Toxicol. 2014; 10: 893–903. DOI: 10.1517/17425255.2014.908185 24793403

[138] Musselman M, Faden J, Citrome L. Asenapine: an atypical antipsychotic with atypical formulations. Ther. Adv. Psychopharmacol. 2021; 11. DOI: 10.1177/20451253211035269 PMC8442490 34540197

[139] Szegedi A, et al. Randomized, Double-Blind, Placebo-Controlled Trial of Asenapine Maintenance Therapy in Adults With an Acute Manic or Mixed Episode Associated With Bipolar I Disorder. Am. J. Psychiatry. 2018; 175: 71–79. DOI: 10.1176/appi.ajp.2017.16040419 28946761

[140] Pahwa M, Sleem A, Elsayed OH, Good ME, El-Mallakh RS. New Antipsychotic Medications in the Last Decade. Curr. Psychiatry Rep. 2021; 23: 87. DOI: 10.1007/s11920-021-01298-w 34843030

[141] Popovic D, Nuss P, Vieta E. Revisiting loxapine: a systematic review. Ann. Gen. Psychiatry. 2015; 14: 15. DOI: 10.1186/s12991-015-0053-3 25859275 PMC4391595

[142] Heffernan MLR, et al. Ulotaront: A TAAR1 Agonist for the Treatment of Schizophrenia. ACS Med. Chem. Lett. 2021; 13: 92–98. DOI: 10.1021/acsmedchemlett.1c00527 35047111 PMC8762745

[143] Koblan KS, et al. A Non–D2-Receptor-Binding Drug for the Treatment of Schizophrenia. N. Engl. J. Med. 2020; 382: 1497–1506. DOI: 10.1056/NEJMoa1911772 32294346

[144] Davidson M, et al. Efficacy and Safety of MIN-101: A 12-Week Randomized, Double-Blind, Placebo-Controlled Trial of a New Drug in Development for the Treatment of Negative Symptoms in Schizophrenia. Am. J. Psychiatry. 2017; 174: 1195–1202. DOI: 10.1176/appi.ajp.2017.17010122 28750582

[145] Shekhar A, et al. Selective Muscarinic Receptor Agonist Xanomeline as a Novel Treatment Approach for Schizophrenia. Am. J. Psychiatry. 2008; 165: 1033–1039. DOI: 10.1176/appi.ajp.2008.06091591 18593778

[146] Watson J, et al. Functional effects of the muscarinic receptor agonist, xanomeline, at 5-HT1 and 5-HT2 receptors. Br. J. Pharmacol. 1998; 125: 1413–1420. DOI: 10.1038/sj.bjp.0702201 9884068 PMC1565721

[147] Brannan SK, et al. Muscarinic Cholinergic Receptor Agonist and Peripheral Antagonist for Schizophrenia. N. Engl. J. Med. 2021; 384: 717–726. DOI: 10.1056/NEJMoa2017015 33626254 PMC7610870

[148] Huhn M, et al. Comparative efficacy and tolerability of 32 oral antipsychotics for the acute treatment of adults with multi-episode schizophrenia: a systematic review and network meta-analysis. Lancet Lond. Engl. 2019; 394: 939–951. DOI: 10.1016/S0140-6736(19)31135-3 PMC6891890 31303314

[149] Schneider-Thoma J, et al. Comparative efficacy and tolerability of 32 oral and long-acting injectable antipsychotics for the maintenance treatment of adults with schizophrenia: a systematic review and network meta-analysis. Lancet Lond. Engl. 2022; 399: 824–836. DOI: 10.1016/S0140-6736(21)01997-8 35219395

[150] Leslie DL, Rosenheck R. Off-label use of antipsychotic medications in Medicaid. Am. J. Manag. Care. 2012; 18: e109–117. 22435962

[151] Leslie DL, Mohamed S, Rosenheck RA. Off-label use of antipsychotic medications in the department of Veterans Affairs health care system. Psychiatr. Serv. Wash. DC. 2009; 60: 1175–1181. DOI: 10.1176/ps.2009.60.9.1175 19723731

[152] Painter JT, et al. Analysis of the Appropriateness of Off-Label Antipsychotic Use for Mental Health Indications in a Veteran Population. Pharmacotherapy. 2017; 37: 438–446. DOI: 10.1002/phar.1910 28164355

[153] Citrome L. Cariprazine for acute and maintenance treatment of adults with schizophrenia: an evidence-based review and place in therapy. Neuropsychiatr. Dis. Treat. 2018; 14: 2563–2577. DOI: 10.2147/NDT.S159704 30323605 PMC6179724

